# Multi-drug resistance of blood stream, urinary tract and surgical site nosocomial infections of *Acinetobacter baumannii* and *Pseudomonas aeruginosa* among patients hospitalized at Felegehiwot referral hospital, Northwest Ethiopia: a cross-sectional study

**DOI:** 10.1186/s12879-020-4811-8

**Published:** 2020-01-30

**Authors:** Hilina Motbainor, Fetlework Bereded, Wondemagegn Mulu

**Affiliations:** Department of Medical Laboratory Science, College of Medicine and Health Sciences, Bahir Dar University, Bahir Dar, Ethiopia

**Keywords:** NIs, MDR, *A. baumannii*, *P. aeruginosa*, Ethiopia

## Abstract

**Background:**

Multi-drug resistant (MDR) *Acinetobacter baumannii* and *Pseudomonas aeruginosa* are major causes of nosocomial infections globally. They are the current World Health Organization critical priority pathogens for resistance, Antimicrobial resistance (AMR) surveillance and discovery of new antibiotics. However, there is paucity of data on nosocomial infections (NIs) caused by such superbugs in Ethiopia. Therefore, this study determined the magnitude and profile of nosocomial MDR *A*. *baumannii* and *P. aeruginosa* infections among patients hospitalized at Felegehiwot referral hospital, Northwest Ethiopia.

**Methods:**

A cross-sectional study was conducted at Felegehiwot referral hospital from April 1 to July 31, 2018. A total of 238 patients with blood stream, urinary tract and surgical site NIs were enrolled conveniently. Either blood, urine and wound swab specimens were collected and processed using standard bacteriological procedures. *A*. *baumannii* and *P. aeruginosa* isolates were identified using standard bacteriological techniques and confirmed by automated Vitek2 Compact. Antimicrobial susceptibility testing on isolates was performed using the disk diffusion technique. The results were interpreted as per the standard zone sizes of Clinical and Laboratory Standards Institute.Chi-square test was done to determine associations among variables. *P* value < 0.05 was considered statistical significant.

**Results:**

The median age of participants was 29 years. Overall,20(8.4%) of patients had nosocomial MDR *A*. *baumannii* and *P. aeruginosa* infections. The proportion of nosocomial MDR blood stream, urinary tract and surgical site infections were 13(8.9%), 5(8.3%) and 2 (6.3%), respectively. Patients with NI had lower mean age (24.9 years) (*P* = 0.035). All isolates of NIs were from patients with intravenous catheterization. The frequency of NI was 9(3.8%) for MDR *A. baumannii* and 11(4.6%) for MDR *P.aeruginosa. A. baumannii* and *P. aeruginosa* isolates were 100% MDR. All isolates of *A. baumannii* and *P. aeruginosa* were 100% resistant to ampicillin and piperacillin.*A. baumannii* isolates were 33.3 and 44.5% resistance against meropenem and ciprofloxacin, respectively while *P.aeruginosa* isolates revealed 36.4 and 45.5% resistance against ciprofloxacin and meropenem, respectively*.*

**Conclusions:**

Health care associated infections of MDR *A*.*baumannii* and *P. aeruginosa* are critical problems in the study area. Therefore, urgent focused interventions required to contain the spreading of MDR NIs. Treatment of NIs for patients on health care should be guided by antimicrobial susceptibility testing.

## Background

Multidrug-resistant (MDR) gram-negative bacterial infections are recognized as one of the major threats to global health. They are leading causes of nosocomial infections in the world [1–3]. NI is an infection occurring in a patient at the time of care in a hospital that was not manifest or incubating during admission but developed after 48 h of hospitalization [4].

The hospital environment contains a large number of immuno-compromised individual’s and patients with diverse bacterial pathogens and normal microbiota [1, 5]. NI accounted 7–10% prevalence in the world [5]. According to 2014 World Health Organization (WHO) report, 15% of all the hospitalized patients suffered from NIs [6].

Surgical site, blood stream and urinary tract infections are the most frequently reported types of NIs [1, 7]. Urinary tract infection (UTI) which involves the bladder, ureter and kidney is the most common type of NIs accounting, 31% [8]. UTI caused by *A*. *baumannii* and *P. aeruginosa* are usually hospital acquired and related to urinary tract catheterization or surgery [9, 10]. Surgical site infection (SSI) can involve the skin, subcutaneous tissue of the incisions, deep soft tissues of the incisions and any part of organ and spaces and accounted for 17% of NIs [8, 10]. Blood stream infection is also one of the common NIs next to UTI and SSIs [10]. It can be acquired from catheter, secondary to another infection site, invasive diagnostic procedures or foreign body [10].

Nosocomial infections with MDR organisms are major global health issues [1–3]. They are very difficult for treatment and main causes of poor clinical outcome, morbidity, mortality, prolonged hospitalization and high health care costs [11, 12]. The situation is true and urgent in Ethiopia.

The non-fermentative gram negative bacilli *A*. *baumannii* and *P. aeruginosa* have emerged as serious particular concern [13, 14]. They are among the most common and serious MDR pathogens documented along with *Enterococcus faecium*, *Staphylococcus aureus*, *Klebsiella pneumoniae* and *Enterobacter* spp., collectively acronyms and considered as ESKAPE pathogens and superbugs, respectively [14, 15].

Multi-drug resistant *A*. *baumannii* and *P. aeruginosa* survive in the hospital setting, transmitted easily between patients through the hands of health care workers [2, 14]. Earlier findings elsewhere in the world reported that *A*. *baumannii* and *P. aeruginosa* commonly possess inherent resistance to antimicrobial agents through reduced permeability of the outer membrane, efflux pump systems, enzymatic inactivation and biofilm formation [15, 16]. Thus, they are often resistant to almost all β-lactams, aminoglycosides and quinolones [15, 17].

Nosocomial isolates of MDR *A. baumannii* and *P. aeruginosa* complicated the treatment of infections and had adverse effect on clinical outcomes and increases patient treatment costs [18]. Factors such as antimicrobial drug overuse, prescription of drugs without susceptibility testing, self- medication and long duration of hospitalization are reported to the occurrence of MDR [11, 19]. However, there is a scarcity of data on the burden of nosocomial MDR *A. baumannii* and *P. aeruginosa* infections in Ethiopia in general and study area in particular. Unavailability of local antibiogram data linked with self-drug prescription by patients and poor awareness on antimicrobial resistance are also big issues. Thus, the study aimed at determining the proportion of nosocomial MDR *A*. *baumannii* and *P. aeruginosa* infection among patients clinically diagnosed for NIs at Felegehiwot referral Hospital (FHRH), Ethiopia.

## Methods

### Study design, period and setting

A cross-sectional study was conducted from April 1 to July 31, 2018 at FHRH, Bahir Dar, Northwest Ethiopia. Bahir Dar is a capital city of the Amhara National Regional State, located approximately 565 km northwest of Addis Ababa. FHRH is a tertiary hospital that provides services for 5 to 7 million people. It has 684 health care and 166 administrative workers. The hospital has 466 beds for inpatient services. FHRH consists of 13 different wards including Surgical, Medical, Gynaecology and Intensive Care Units (ICUs). It has adult and neonatal ICUs with 8 beds. Bacteriological procedures were conducted at FHRH microbiology laboratory and further confirmatory identification of isolates and screening of MDR made using VITEK2 compact at Amhara Public Health Institute (APHI), Ethiopia. All patients admitted at FHRH and clinically presumptive for either of nosocomial urinary tract, post-operative surgical site and blood stream infection during the study period were the study population.

### Sample size and sampling

The sample size for NIs was determined using Epi info version 3.5.1 (public domain software, www.cdc.gov) by considering 95% confidence level and marginal error (5%). A proportion of 0.83 MDR NIs taken from previous study in other parts of South East Ethiopia [16]. Thus, the total sample size was 238. All patients who were clinically presumptive for nosocomial blood stream, urinary tract and post-operative surgical site nosocomial infections were included conveniently until the required sample size was achieved.

### Variables

Multi-drug resistant blood stream, urinary tract and surgical site nosocomial infections *A. baumannii* and *P. aeruginosa* were the dependent variables while demographic variables (age, sex, residence, educational status, occupation) and clinical data on co-morbidity, urinary catheterization, intravenous catheterization, duration of catheterization, previous history of antibiotics, previous history of surgery, duration of hospitalization, wards of patients hospital and duration of operation were the independent variables.

### Inclusion and exclusion criteria

All patients who had either clean and clean-contaminated operations or other medical reasons admitted in Medical, Surgical and ICU wards of FHRH and developed clinical evidences of blood stream, urinary tract and surgical site nosocomial infections after 48 h of admission were included during the study period. However, patients who had re-operation, contaminated and dirty operations, ventilator- associated pneumonia, skin infections and developed any type of clinical evidences of infection within 48 h of admission were excluded from the study.

### Data collection

Information on demographic variables was collected from each participant by face-to-face interview using a structured questionnaire. Clinical data related to co-morbidities, hospitalization, surgery and use of antibiotics were collected by reviewing patient’s medical record and in consultation with the respective physician. With the study team, patients admitted in the Medical, Surgical and ICU wards were carefully diagnosed for NIs by Internists and Surgeon. Clinical specimens (blood, urine and wound swab) were collected by the study team as soon as NI was reported following the bacteriological standard procedures.

The criteria of The European Centre for Disease Prevention and Control were used to define NIs [10]. Patients who had either purulent drainage, pain, localized swelling, redness, or heat in the skin, subcutaneous tissue, deep soft tissue, organ or spaces and one positive culture for *A*. *baumannii* and *P. aeruginosa* after 48 h of operation were considered as nosocomial surgical site infection. Patients who had either fever (> 38 °C), urgency, frequency, dysuria, or suprapubic tenderness with no other recognized cause but has ≥10^2^ CFU/milliliters (ml) and ≥ 105 CFU/ml of urine culture for catheterized and non- catheterized patients, respectively after 48 h of admission were considered as nosocomial urinary tract infection. On the other hand, patients who had either fever (> 38 °C), chills, or hypotension and one positive blood culture for *A. baumannii* and *P. aeruginosa* after 48 h of admission were considered as nosocomial blood stream infection.

### Wound swab collection and processing

Two wound samples from each participant were collected aseptically by sterile cotton swabs dipped in normal saline using Levine method [20]. The wound swabs were inoculated on MacConkey agar (MAC) and Blood agar (BA) (Oxoid, UK) at a time. Both plates were incubated at 37 °C and examined for visible bacterial growth after 48 h of incubation.

### Blood sample collection and processing

As per the standard protocol, 1, 2 and 10 ml of venous blood were collected from neonates, children and adults presumptive for blood stream infection, respectively [21]. Following cleaning of the site of blood collection with 70% alcohol and 2% tincture iodine, two blood samples were collected from two different sites of peripheral vein of each febrile patient using two bottles of blood sample within 30 min difference. The collected blood samples were inoculated directly to 5 - 10 ml Tryptic Soya broth blood culture medium bottle (Oxoid, UK) and transported to FHRH microbiology laboratory. All blood culture broths were incubated aerobically at 37 °C and regular subcultures were done on BA and MAC (Oxoid, UK) after 1, 2 and then 3 days later daily up to 7 days of incubation. All of the inoculated agar plates were incubated at 37°c. Finally, plates were examined for bacterial growth after 24 h.

### Urine sample collection and processing

Clean-catch mid-stream urine was collected from patients presumptive for UTI. From catheterized patients, 5 ml of catheterized urine transferred to a sterile container after cleansing the out let of catheter. For non-catheterized patients, the same amount of urine sample was collected by the patient using sterile container. Urine samples were inoculated on MAC and BA (Oxoid, UK). All agar plates were incubated aerobically at 37 °C for 24 h and observed for bacterial growth. Blood agar colonies were counted using colony counter and checked for significant bacteriuria. Culture from catheterized and non-catheterized patients that grew ≥10^2^ CFU/ml and 10^5^ CFU/ ml, respectively was taken as a significant bacteriuria, respectively. For heterogeneous colonies, sub-culturing of individual distinct colonies was performed to ensure pure cultures.

### Identification of bacterial isolates

All isolates of *A*. *baumannii* and *P. aeruginosa* were identified by manual standard microbiological methods [21]. All *A.baumannii* and *P. aeruginosa* suspected isolates were also further confirmed by an automated Vitek2 Compact (BioMérieux, France).

### Antimicrobial susceptibility testing

Antimicrobial susceptibility testing was carried out for each isolates of *A*. *baumannii* and *P. aeruginosa* on Mueller Hinton agar (MHA) (Oxoid, UK) by Kirby - Bauer disk diffusion technique [22]. All *A*. *baumannii* and *P. aeruginosa* isolates were tested against the following classes of antibiotics: Penicillin (ampicillin (10 μg)), piperacillin (100 μg)), β-lactam/β-lactamase inhibitor combination (amoxicillin-clavulanic acid (20/10 μg)), cephalosporin (ceftazidime (30 μg), cefotaxime (30 μg) and ceftriaxone (30 μg)), aminoglycosides (gentamycin (10 μg)), fluoroquinolones (ciprofloxacin (10 μg)), Folate pathway inhibitor (trimethoprim-sulphamethoxazole(1.25/23.75 μg)), carbapenem (meropenem (10 μg)) and tetracycline (30 μg) (Oxoid, England). The results were interpreted using the standard zone sizes of the Clinical and Laboratory Standards Institute (CLSI, 2017) guidelines [23].

### Quality control

The prepared questionnaire was checked for its completeness and validity prior to the data collection. All the standard operating procedures (SOPs) were strictly followed at all stages of microbiological analysis. American Type Culture Collection (ATCC) standard reference strains (*P. aeruginosa* ATCC27853 and *E. coli* ATCC 25922) were used for quality control of antimicrobial susceptibility testing. MDR *A*. *baumannii* and *P. aeruginosa* isolates were confirmed by using Vitek2 compact. A standardized bacteriological procedure was followed to maintain correct laboratory results. At regular intervals and whenever a new batch of strain or reagent is prepared, standard strains of *P. aeruginosa* ATCC27853 and *E. coli* ATCC 25922 were used as positive controls. The sterility of the media was checked by incubating the media overnight before its use. The data were checked for completeness and representativeness prior to entry.

### Data analysis

Data were checked, entered and analyzed using Statistical Package for Social Science 23 (IBM Corp Released 2011.IBM SPSS statistics. Armonk, NY: IBM Corp). Descriptive statistics were used to describe relevant variables. Chi-square test, Fishers exact test and Independent samples T Test was obtained to determine association between dependent and independent variables. *P* value of < 0.05 was considered statistical significant.

## Results

### Demographic characteristics

A total of 238 patients with clinical evidence of nosocomial infection (BSI, UTI and SSI) were enrolled in the study. Of them, 129 (54.2%) were males. The majority (21.4%) of participants were found in the age group of > 51 years with median age of 29 years. One hundred twenty six (52.9%) of the study participants were from urban settings. Data on occupation showed that majority (39.1%) of participants were government employee. Table 1 depicts the demographic characteristics of the study participants**.**

**Table 1:** Demographic characteristics of patients clinically presumptive for nosocomial infection at FHRH, 2018 (*n* = 238)

Demographic variables	Number	Percent
Sex		
Female	109	45.8
Male	129	54.2
Age (in years)		
< 1	31	13.0
1–10	34	16.4
11–20	29	12.2
21–30	47	19.7
31–40	25	10.5
41–50	21	8.8
> 51	51	21.4
Residence		
Urban	126	52.9
Rural	112	47.1
Occupation		
Farmer	23	15.2
House wife	31	20.5
Government employee	59	39.1
Private employee	27	17.9
Other work	11	7.3
Unemployed	87	36.6
Education		
Under school age	49	20.6
Illiterate	52	27.5
Elementary completed	60	31.7
Highschool completed	11	5.8
Diploma	36	19.0
Degree and above	30	15.9

Key: Unemployed: Below school age + students; Other work: daily laborer and merchant

### Rate of nosocomial infection and frequency of bacterial isolates

The overall prevalence of the combined nosocomial MDR *A*. *baumannii* and *P. aeruginosa* infection was 20 (8.4%). Of them, the proportion of BSI, UTI and SSI were 13 (8.9%), 5 (8.3%) and 2 (6.3%), respectively. The proportion of nosocomial MDR *A*. *baumannii* and *P. aeruginosa* infection were 9 (3.8%) and 11 (4.6%), respectively. *P. aeruginosa* accounted for 6.3, 4.8 and 3.3% of nosocomial SSI, BSI and UTI, respectively while, MDR *A*. *baumannii* causes 5 and 4.1% of nosocomial UTI and BSI, respectively (Table 2).

**Table 2:** Proportion of nosocomial MDR *A*. *baumannii* and *P. aeruginosa* infection in patients clinically presumptive for nosocomial blood stream, urinary tract and surgical site infection at FHRH, 2018 (*n* = 238)

Type of MDR isolates	Rate of nosocomial infection N (%)			
	UTI (*n* = 60)	SSI (*n* = 32)	BSI (*n* = 146)	Total
*A. baumannii*	3 (5)	0 (0)	6 (4.1)	9 (3.8)
*P. aeruginosa*	2 (3.3)	2 (6.3)	7 (4.8)	11 (4.6)
Total	5 (8.3)	2 (6.3)	13 (8.9)	20 (8.4)

Participants with NIs had lower mean of age (24.9 years) than those without NIs (29.6 years) and the difference was statistical significant (*P* = 0.035). Highest (15.4%) NIs rate was found in age groups < 10 years. The proportion of NI was 13 (11.4%) in those participant with co-morbidity. All isolates of NIs were from patients with intravenous catheterization (Table 3). Duration of operation was significantly higher in those patients with confirmed NIs (180 min) than their counterparts (155 min) (*P* = 0.04). Moreover, the duration of catheterization was higher in those with confirmed NIs (13.6 days) than their counter parts (11.3 days). However, the difference was not statistical significant (*P* = 0.25) (Table 3).

**Table 3:** Distribution of nosocomial MDR *A*. *baumannii* and *P*. *aeruginosa* infection in different variables of study participants clinically presumptive for nosocomial infection at FHRH, 2018 (*n* = 238)

Variables		Confirmed rate of Nosocomial infection			*P*-value
		Positive N (%)	Negative. N (%)	Total. N (%)	
Sex	Female	9 (8.3)	100 (91.7)	109 (45.8)	
	Male	11 (8.5)	118 (91.5)	129 (54.2)	0.94
Age	< 1	5 (16.1)	26 (83.9)	31 (13.0)	
	1–10	5 (14.7)	29 (85.3)	34 (16.4)	
	11–20	1 (3.4)	28 (96.6)	29 (12.2)	0.34
	21–30	2 (4.3)	45 (95.7)	47 (19.7)	
	31–40	2 (8)	23 (92)	25 (10.5)	
	41–50	0 (0)	21 (100)	21 (8.8)	
	> 51	5 (9.8)	46 (90.2)	51 (21.4)	
Residence	Urban	9 (7.1)	117 (92.9)	126 (52.9)	
	Rural	11 (9.8)	101 (90.2)	112 (47.1)	0.46
Education	Non-educated	3 (5.8)	49 (94.2)	52 (27.5)	
	Educated	8 (5.8)	129 (94.2)	137 (31.7)	
	Below school age	9 (18.4)	40 (81.6)	49 (20.6)	1.00
Type of NI	UTI	5 (8.3)	55 (91.7)	60 (25.2)	
	SSI	2 (6.3)	30 (93.8)	32 (13.4)	
	BSI	13 (8.9)	133 (91.1)	146 (61.3)	
Occupation	Farmer	0 (0)	23 (100)	23 (15.2)	
	House wife	4 (12.9)	27 (87.1)	31 (20.5)	0.20
	Government employee	5 (8.5)	54 (91.5)	59 (39.1)	
	Private employee	0 (0)	27 (100)	27 (17.9)	
	Other	1 (9.1)	10 (90.9)	11 (7.3)	
	Unemployed	10 (11.5)	77 (88.5)	87 (36.6)	
Ward of patients hospital	Surgical	4 (8.5)	43 (91.5)	47 (19.7)	
	Medical	11 (8.1)	125 (91.9)	136 (80.3)	
	ICU	5 (9.1)	50 (90.9)	55 (23.1)	0.48
Underlying disease	Yes	13 (11.4)	101 (88.6)	114 (47.9)	0.11
	No	7 (5.6)	117 (94.4)	124 (52.1)	
Urinary Catheterization	Yes	9 (7.4)	112 (92.6)	121 (50.8)	
	No	11 (9.4)	106 (90.6)	117 (49.2)	0.59
Intravenous Catheterization	Yes	20 (9.0)	201 (91.0)	221 (92.9)	0.37
	No	0 (0)	17 (100)	17 (7.1)	
Previous antibiotics	Yes	14 (9.0)	141 (91.0)	155 (65.1)	0.633
	No	6 (7.2)	77 (92.8)	83 (34.9)	
Previous surgery	Yes	5 (10.2)	44 (89.8)	49 (20.6)	0.61
	No	15 (7.9)	174 (92.1)	189 (79.4)	
Total		20 (8.4)	218 (91.6)	238 (100)	
Mean Age of participants		24.9	29.6		0.035
Duration of hospitalization (days)		14.5	15.7		0.48
Duration of operation (Minutes)		180	155		0.04
Duration of catheterization (Days)		13.6	11.3		0.25

### Multi-drug resistance profiles of *A*. *baumannii* and *P. aeruginosa* isolates

*A*. *baumannii* and *P. aeruginosa* isolates were resistant to three to six antibiotics from different classes. All isolates of *A*. *baumannii* (9) and *P. aeruginosa* (11) were MDR (100%). Among this, 3 (33.3%) isolates of *A*. *baumannii* and 4 (36.4%) isolates of *P. aeruginosa* showed resistance to antibiotics from six different classes, respectively. Three (33.3%) isolates of *A*. *baumannii* and 1 (9.1%) isolates of *P. aeruginosa* were resistant against antibiotics from 4 different classes, respectively. Moreover, 3 (33.3%) of isolates of *A*. *baumannii* and 6 (54.5%) of *P. aeruginosa* isolates were resistant against antibiotics from three different classes (Table 4).

**Table 4:** Multi-drug resistance profile of *A*. *baumannii* and *P*. *aeruginosa* isolates from patients clinically presumptive for nosocomial infection at FHRH, 2018 (*n* = 20)

Bacterial isolate	Antibiogram Profile	Antibiotic Class	Frequency N (%)	Over all, MDR
*A*. *baumannii* (*n* = 9)	(AMP,AMC,CAZ,PIP,CIP,CN,SXT,MEM)	6	3 (33.3)	
	(AMP,AMC,CAZ,PIP,CN,SXT)	4	2 (22.2)	
	(AMP,AMC,CAZ,PIP,CIP,CN)	4	1 (11.1)	
	(AMP,AMC,PIP,CN,SXT)	3	2 (22.2)	
	(AMP,CAZ,PIP,SXT)	3	1 (11.1)	
				9 (100)
*P*. *aeruginosa* (*n* = 11)	(AMP,AMC,CAZ,PIP,CIP,CN,SXT,MEM)	6	4 (36.4)	
	(AMP,AMC,CAZ,PIP,CIP,SXT)	4	1 (91.)	
	(AMP,AMC,PIP,CN,SXT)	3	2 (18.2)	
	(AMP,AMC,CAZ,PIP,SXT)	3	4 (36.4)	
				11 (100)

Key: AMP: Ampicillin, AMC: Amoxicillin-clavulanic acid, PIP: Piperacillin, CAZ: Ceftazidime, CIP: Ciprofloxacin, CN: Gentamicin, MEM: Meropenem and SXT: Sulphamethoxazole- Trimethoprim, R3,4,6,: resistance to 3,4,6 antibiotic drug classes

### Antimicrobial resistance profiles of *A*. *baumannii* and *P. aeruginosa*

Both *A*. *baumannii* and *P. aeruginosa* isolates showed 100% resistance against ampicillin and piperacillin. Besides, *P. aeruginosa* isolates showed 100% resistance against amoxicillin-clavulanic acid, cefotaxime, ceftriaxone and trimethoprim-sulphamethoxazole. *A*. *baumannii* isolates showed resistance 88.9% to amoxicillin-clavulanic acid, ceftriaxone and cefotaxime and also 77.8% to tetracycline and ceftazidime. However, *P. aeruginosa* isolates revealed a high resistance rate to ceftazidime (63.6%) and tetracycline (90.6%). *A*. *baumannii* isolates showed low level resistance rate to ciprofloxacin (44.5%) and meropenem (33.3%). Similarly, *P. aeruginosa* isolates showed low level of resistance against ciprofloxacin (36.4%) and meropenem (45.5%) (Table 5).

**Table 5:** Antimicrobial resistance profiles of *A*. *baumannii* and *P*. *aeruginosa* isolates from participants presumptive for nosocomial infection at FHRH, Bahir Dar, Northwest Ethiopia, April to July, 2018

Antimicrobials	*A.baumannii*		*P*.*aeruginosa*		Total	
	# T	R %	# T	R %	# T	R%
Ampicillin	9	9 (100)	11	11 (100)	20	20 (100)
Amoxicillin-clavulanic acid	9	8 (88.9)	11	11 (100)	20	19 (95)
Piperacillin	9	9 (100)	11	11 (100)	20	20 (100)
Cefotaxime	9	8 (88.9)	11	11 (100)	20	19 (95)
Ceftriaxone	9	8 (88.9)	11	11 (100)	20	19 (95)
Ceftazidime	9	7 (77.8)	11	7 (63.6)	20	14 (70)
Ciprofloxacin	9	4 (44.5)	11	4 (36.4)	20	8 (40)
Gentamicin	9	8 (88.9)	11	6 (54.5)	20	14 (70)
Meropenem	9	3 (33.3)	11	5 (45.5)	20	8 (40)
Tetracycline	9	7 (77.8)	11	10 (90.9)	20	17 (85)
Sulphamethoxazole - Trimethoprim	9	6 (66.7)	11	11 (100)	20	17 (85)
Total	99	77 (77.8)	121	98 (80.99)	220	175 (79.5)

#T: Number of isolates tested, R%: Percentage of resistant isolates

## Discussion

Antibiotic resistant nosocomial infections are becoming serious health care problem in ICU and other areas of hospital care, increasing morbidity, mortality, length of stay, and health care costs [2, 24]. The epidemiological and antimicrobial resistance profiles of NIs showed variations among hospitals around the globe. Many of the infections are caused by bacteria that are resistant to multiple antibiotics [24, 25]. This study showed the proportion of NIs due to two MDR non-fermentative gram negative bacilli among patients hospitalized in different wards of a referral hospital.

In the present study, 8.4% of patients were infected with nosocomial MDR *A*. *baumannii* and *P. aeruginosa*. This indicated that MDR *A*.*baumannii* and *P. aeruginosa* infections are the major health problem in the clinical area in Ethiopia. High patient load, overcrowding, poor infrastructure, poor infection control practices of the hospital and differences in trained medical staff for aseptic procedures might be the possible explanations. This finding was coherent with reports in Tikur Anbessa Hospital, Ethiopia (8.12%) [25], Uganda (7.39%) [26], Italy (9.3%) [27] and Gaza city (6.9%) [28]. However, it was higher compared to reports from Hiwot Fana Hospital, Ethiopia (0.5%) [8], Gabon (5.7%) [29], China (0.78%) [30] and Indonesia (3.5%) [31]. In contrast, the overall nosocomial MDR *A*. *baumannii* and *P. aeruginosa* infections in the present study was lower than studies done in Ghana (23.5%) [32]. This might be due to variation in sample size, clinical site of infection, age of patients, hospital setting, duration of hospitalization, patients exposure to high risk devices or surgical procedures, microbiological methods employed for screening of MDR resistant strains.

The highest proportion of MDR non-fermentative gram negative bacilli NI infection among the lower age groups in the present study is consistent with earlier studies elsewhere in the world [29, 33, 34]. On the other hand, in the present study all NIs observed among patients with intravenous catheterization. The rate of nosocomial infections was also significantly higher among patients who had prolonged time of operation than their counter parts. This was consistent with previous study in Tikur Anbessa Hospital, Ethiopia (25). This might be due to the high rate of exposure of patients to the two MDR pathogens from the hospital environment, health care professionals, multiple invasive device and cross-contamination among patient’s procedures.

In this study, the proportion of nosocomial MDR *A*. *baumannii and P. aeruginosa* surgical site infection (6.3%) was comparable with previous reports from other part of Ethiopia (6.6%) [25] and Ghana (8.5%) [32]. However, it was lower than studies from Southeast China (28.5%) [35]. This could be the difference in the type of surgery, handling of surgical equipments and age of study participants as the present study included patients of any age groups.

The proportion of nosocomial urinary tract infection linked with MDR *A*. *baumannii* and *P. aeruginosa* (8.3%) isolates in the present study was consistent with a study conducted in Kenya (9%) [36]. However, it was lower than studies in Gabon (52.8%) [29], Indonesia (16.5%) [31] and United States of America (USA) (16%) [37]. This could be due to differences among study participants, catheterization and hospitalization.

The prevailing proportion of nosocomial BSI in this study (8.9%) was comparable with studies done in USA (10%) [37]. However, it was higher than studies from Indonesia (3.5%) [31]. In contrast, lower findings were documented elsewhere in Africa (20–70.3%) [29, 38] and Southeast China (46.1%) [35]. The observed difference might be due to non-sustainable infection control practices in hospitals, difference in use of invasive medical devices, procedures, hospital type and diverse nature of study participants.

The proportion of nosocomial MDR *A*. *baumannii* infection (3.8%) in the present study was in agreement with earlier studies conducted in Africa (2.4–5.7%) [26, 29, 32]. However, it was lower compared with findings from Sodo (15.3%), Ethiopia [18] and Gaza city (6.9%) [28]. In contrast, it was higher than findings from previous studies in elsewhere (0.42–0.95%) [27, 30]. Similarly, the magnitude of nosocomial MDR *P. aeruginosa* infection (4.6%) in the present study was comparable with a study in Uganda (5%) [26]. However, it was lower than findings from other parts of Ethiopia (11.1–66.7%) [8, 18, 25], Ghana (19.5%) [32], India (76.8%) [39] and Italy (8.7%) [27]. In contrast, it was higher compared to a study in China (0.36%) [30]. The variations might link with host, microbial and environmental factors.

In this study all isolates of MDR *P. aeruginosa* were resistant for ampicillin and amoxacillin-clavulanic acid. This was consistent with reports from Tikur Anbessa hospital, Ethiopia (25) and Southeast China [35] where 87.5 and 100% resistance rate against ampicillin and amoxacillin-clavulanic acid respectively were noticed. Moreover, all isolates of *P. aeruginosa* in the present study were resistant against piperacillin. This was significantly higher than studies from Italy (25%) [27], Southeast China (12%) [35], Turkey (28.7%) [40] and Taiwan (66.8%) [41]. This might be associated with differences in the number of MDR strains of *P. aeruginosa* and patient type. The frustrating level of resistance against piperacillin antibiotic is an alarm for treatment to be guided with antimicrobial susceptibility testing as it was not prescribed in the study area (FHRH).

In this study, high levels of resistance to cephalosporins (cefotaxime (63.6%) and ceftazidime (100%)) were obtained against *P. aeruginosa* isolates. This was coherent with studies in Uganda [23], India [39] and Taiwan [41] where 71–77% resistance against ceftazidime reported. Moreover, 70.8 and 92.8% level of resistance against cefotaxime were documented in Sodo, Ethiopia [18] and Southeast China [35], respectively. On the other hand, low level of resistance against ceftazidime reported in other parts of Ethiopia (12.5–29.1%) [18, 25] and Italy (31%) [27]. The highest level of resistance against third generation cephalosporins might be linked with excessive, mis and inappropriate use of these antibiotics in the area that drives selective pressure and emergence of MDR.

The resistance rate of *P. aeruginosa* isolates against meropenem (45.5%) in the present study was coherent with other studies in Ethiopia (41.7%) [18] and Asia (36.6–54%) [33, 39]. However, higher level of resistance against meropenem was documented in Taiwan (73.2%) [41] and Saudi (81.8%) [42]. The relatively lower proportion resistance against meropenem in the present study might be linked with the nonexistence of meropenem prescription practice in the study hospital. In contrast, lower level of resistance against meropenem was reported in Uganda (14%) [26] and Turkey (20.4%) [40]. This could be due to variation in the availability of meropenem in each localities, prescription difference, misuse and inappropriate use of antibiotics.

In this study, all isolates of MDR *A*. *baumannii* were resistance against ampicillin. This was parallel with studies conducted in Tikur Anbessa Hospital (88.2%), Ethiopia [25] and Southeast China (100%) [35]. Moreover, high level (88.9%) of MDR *A*. *baumannii* isolates resistance to amoxacillin clavulanic acid in the present study was comparable with earlier studies in Nigeria [43] and Southeast China [35], where all isolates of *A*. *baumannii* were resistant against amoxacillin- clavulanic acid.

All isolates of *A. baumannii* were resistant to piperacillin in the present study. This was comparable with studies conducted in Southeast Asia (83.7%) [35] and Italy (81) [27]. Similarly, *A. baumannii* isolates also revealed 77.8 and 88.9% resistance against ceftazidime and cefotaxime, respectively which was comparable with studies done in Tanzania [44], Southeast China [35] and Italy [27], where resistance against ceftazidime and cefotaxime reported in 71–97.1% and 54.4–100% of isolates, respectively. Furthermore, 33.3% of *A*. *baumannii* isolates from the present findings showed resistance against meropenem. The finding was relatively similar with studies conducted in Sodo, Ethiopia [18], Tanzania [44] and Nigeria [43] where, 30.2–40% resistance rate against meropenem reported. However, in Saudi [42], resistance against meropenem was reported in 90.5% of *A. baumannii* isolates*,* respectively.

The resistance level of MDR isolates of *A. baumannii* to ciprofloxacin (44.5%) in the present study was lower than studies done in Tikur Anbessa Hospital (70.6%) and Sodo (88.4%), Ethiopia [18, 25], Uganda (78%) [26], Nigeria (100%) [43], Southeast China (89.6%) [35] and Italy (84%) [27]. The low resistance rate against ciprofloxacin and meropenem in the present study might be due to the high price and unavailability of the drugs in the hospital.

In this study, all isolates of *A*. *baumannii* and *P. aeruginosa* were MDR (100%). This finding was consistent with studies conducted in Sodo (81.4 & 83.3%), Ethiopia [18], Tanzania (100%) [44], Ghana (100%) [30] and India (76.8%) [43]. However, it was higher than reports from Uganda (38 & 40%) [26], Saudi (28.2%) [42], Saudi Arabia (71.8%) [45] and Italy (20 & 54%) [27]. Therefore, this very alarming MDR proportion of *A*. *baumannii* and *P. aeruginosa* isolates in the study area needs urgent intervention and strict adherence to infection control practices to contain them.

The high MDR proportion observed in two non-fermenter gram negative bacilli in this study is probably related to the contaminations and cross transmission of these bacteria from hospital environment [18], hands of healthcare workers, frequent use of broad spectrum antibiotics, inherent resistance nature to many antimicrobial agents and the ability of pathogens persist in the environment, and on medical devices for a longer time [2]. Moreover, high prescription practice of common antibiotics including third generation cephalosporins and use of drugs outside the hospital might contribute for the high resistance rate of *A*. *baumannii* and *P. aeruginosa* to different classes of antibiotics.

This study was limited to participants admitted in hospital but nosocomial infection that arose after discharge was not detected. The single-centre nature of the study introduced local practice bias. The study also unable to demonstrate cross-transmission of pathogens and ignored the effect of duration of antibiotic therapy at individual patient level. Moreover, certain potential confounders could not be included in the analysis.

## Conclusions

Alarming proportion of nosocomial MDR *A*. *baumannii* and *P. aeruginosa* infection obtained in the study area. All *A*. *baumannii* and *P. aeruginosa* isolates were resistant for at least three antibiotics representative of different classes. Therefore, urgent intervention towards nosocomial infection prevention practices required and treatment of patients on care should be guided with antimicrobial susceptibility testing.

## Data Availability

The finding of this study is generated from the data collected and analyzed based on the stated methods and materials. All the data are already found in the manuscript and there are no supplementary flies. The original data supporting this finding will be available at any time upon request.

## Abbreviations

AMC: Amoxicillin-clavulanic acid
AMP: Ampicillin
AMR: Antimicrobial resistance
APHI: Amhara Public Health Institute
ATCC: American Type Culture Collection
BA: Blood agar
BSI: Blood Stream Infection
CAZ: Ceftazidime
CFU: Colony Forming Unit
CIP: Ciprofloxacin
CLSI: Clinical and Laboratory Standards Institute
CN: Gentamicin
ESKAPE: (*Enterococcus faecium*, *Staphylococcus aureus*, *Klebsiella pneumoniae*, *Acinetobacter baumannii*, *Pseudomonas aeruginosa*, *Enterobacter* species)
FHRH: Felegehiwot Referral Hospital
ICU: Intensive Care Unit
MAC: MacConkey agar
MDR: Multi-Drug Resistance
MEM: Meropenem
MHA: Mueller- Hinton agar
ml: Milliliters
NI: Nosocomial Infection
PIP: Piperacillin
SOPS: Standard Operating Procedures
SSI: Surgical Site Infection
SXT: Sulfamethoxazole –Trimethoprim
UK: United Kingdom
USA: United States of America
UTI: Urinary Tract Infection
WHO: World Health Organization
